# Insects as a New Complex Model in Hormonal Basis of Obesity

**DOI:** 10.3390/ijms222011066

**Published:** 2021-10-14

**Authors:** Karolina Walkowiak-Nowicka, Szymon Chowański, Arkadiusz Urbański, Paweł Marciniak

**Affiliations:** 1 Department of Animal Physiology and Developmental Biology, Faculty of Biology, Adam Mickiewicz University in Poznań, 61-614 Poznań, Poland; szymon.chowanski@amu.edu.pl (S.C.); arkadiusz.urbanski@amu.edu.pl (A.U.); pawel.marciniak@amu.edu.pl (P.M.); 2 HiProMine S.A., 62-023 Robakowo, Poland

**Keywords:** insects, obesity, model, hormones, neuropeptides, peptides

## Abstract

Nowadays, one of the biggest problems in healthcare is an obesity epidemic. Consumption of cheap and low-quality energy-rich diets, low physical activity, and sedentary work favor an increase in the number of obesity cases within many populations/nations. This is a burden on society, public health, and the economy with many deleterious consequences. Thus, studies concerning this disorder are extremely needed, including searching for new, effective, and fitting models. Obesity may be related, among other factors, to disrupting adipocytes activity, disturbance of metabolic homeostasis, dysregulation of hormonal balance, cardiovascular problems, or disorders in nutrition which may lead to death. Because of the high complexity of obesity, it is not easy to find an ideal model for its studies which will be suitable for genetic and physiological analysis including specification of different compounds’ (hormones, neuropeptides) functions, as well as for signaling pathways analysis. In recent times, in search of new models for human diseases there has been more and more attention paid to insects, especially in neuro-endocrine regulation. It seems that this group of animals might also be a new model for human obesity. There are many arguments that insects are a good, multidirectional, and complex model for this disease. For example, insect models can have similar conservative signaling pathways (e.g., JAK-STAT signaling pathway), the presence of similar hormonal axis (e.g., brain–gut axis), or occurrence of structural and functional homologues between neuropeptides (e.g., neuropeptide F and human neuropeptide Y, insulin-like peptides, and human insulin) compared to humans. Here we give a hint to use insects as a model for obesity that can be used in multiple ways: as a source of genetic and peptidomic data about etiology and development correlated with obesity occurrence as well as a model for novel hormonal-based drug activity and their impact on mechanism of disease occurrence.

## 1. Introduction

With rising problems with the occurrence of overweight and thus obesity/diabetes researchers are looking for causes of those disorders and are focused on describing its course and on finding effective remedies. Therefore, searching for a new disease research model is justified. For many years, for biomedical research, insects have played the role of model organisms. The most studied insect is undoubtedly *Drosophila melanogaster*, but variety of insects which are used as a models might be indicated, for instant *Tribolium castaneum*, *Galleria mellonella*, *Bombyx mori*, *Periplaneta americana*, or *Locusta migratoria* [1,2,3,4].

Worldwide distribution, environmental significance, and the relatively low cost of rearing as a consequence of ease of breeding, short life cycle, low financial outlays, and fewer ethics problems are arguments for using insects as a model for human-related affections and diseases [1]. However, they are not enough. There are at least a few more pros that create insects for usage as models. Choosing insects as a model is justified due to the similarity of their signaling pathways, energy metabolism, and structural components with mammals as well as activity of feedback loops. For example, multiple research projects have showed functional conservation of such hormonal systems like one engaged in homeostatic maintenance of hemolymph sugar level, and insulin/glucagon in which a homolog in insects is described as insulin-like (ILPs)/glucagon-like peptides systems [5,6,7,8,9,10]. Another examples of an analogue of hormonal systems that can be found in vertebrates and insects are glucagon and adipokinetic hormones (AKHs) or tachykinin-related peptides (TRPs) that resemble mammalian tachykinins (TKs) [11].

Functional homology of structures, e.g., fat body tissue and adipose tissue/liver, *corpus cardiacum/corpus allatum* and pituitary gland, *pars intercerebralis*—*pars lateralis* and the hypothalamus is also important [1]. In addition, in insects, several genes correlated with human body weight (e.g., *SH2B1*, *HMGCR* or *TUB*) were identified [12]. Gene *SH2B1* encodes SH2B adapter protein 1, which acts as a regulator of tyrosine kinases, e.g., insulin and insulin-like growth factor-1 (IGF-like) including receptors [12,13]. Among the insect order its homolog *Lnk* was found. Lnk plays a similar role as SH2B1 in insulin and IGF-1 signaling [14,15]. Another example of homology between mammalian and insect gene is *HMGCR* responsible for encoding 3-hydroxy-3-methylglutaryl-coenzyme A reductase (in insects it is also called HMGCR) which in both mammals and insects is responsible for regulation of processes correlated with cholesterol production, as it catalyzes mevalonate, a substrate for cholesterol biosynthesis [16,17]. Mammalian *TUB* gene is related with the occurrence of TUB protein, which belongs to the tubby-like proteins (TULPs), related with maturity-onset obesity formation [18]. In *Drosophila*, the orthologue of TUB protein is known as a king-tubby, and its action is *strict* for autophagy regulation [12].

The next argument for usage of insects as a model for human diseases are similarities between signaling pathways, e.g., Toll/Dif (in which the main role is played by the Toll receptor which resembles the mammalian Toll-like receptor (TLRs), and the Spätzle protein, for which the mammalian analogue is called nerve growth factor (NGF) or interleukin 17F) or Imd/Relish (Relish is a transcription factor belongs to nuclear factors ĸB (NFĸB) which are crucial in mammals for activation tumor necrosis factor (TNF) signaling) [19,20,21]. In addition, the JAK/STAT signaling pathway may be a good example as it plays an important role in insects but was originally described in mammals [11,19,20].

**Figure 1:**
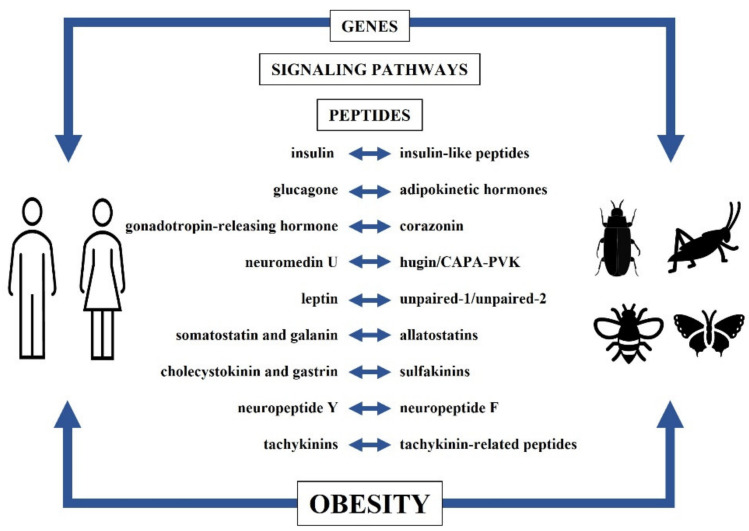
Similarities between human and insects’ factors correlated with obesity occurrence.

There are also similarities in peptides and neuropeptides structures and functions, based on which peptides are often considered or even indicated as a homolog of mammalian or human peptides/neuropeptides [1,11]. Here we bring the subject matter closer, focusing on similarities between insects and human peptides/neuropeptides and their potential possibility of use as a model in overweight/obesity correlated affections in humans (Figure 1). In the case of insect peptides we use the nomenclature of Coast and Schooley [22].

## 2. Peptides–Neuropeptides–Hormones

### 2.1. Insulin-Like Peptides

Insulin is one of the most deeply studied animal peptide hormones [23]. This anabolic hormone elicits metabolic effects throughout the whole body. It regulates glucose and other carbohydrate metabolism as well as lipid and protein usage by cells [24]. Thus, its role in the development of many nutritional disorders, such as obesity, is undeniable. Moreover, insulin is a part of bigger family of peptide hormones, the so-called insulin-like peptide family, which includes, in human, two IGFs, one relaxin, and a number of insulin-like peptides (INSL3-7) [25] which also have a significant impact on metabolism, thus, might be involved in development of obesity. ILPs are not limited to humans, but are widespread in the animal kingdom, including insects [26]. The first insect ILP was purified from adult heads of the silk moth *B. mori* as a peptide that stimulated ecdysone production in another moth species [27]. Later, more ILPs in other insect species were identified, ranging from one in *L. migratoria* to 39 in *B. mori* [28]. However, they can be classified into at least three subfamilies: insulin-like peptides, IGF-like peptides, and Dromo-ILP7-like peptides [29]. The similarities between human and insect ILPs concern a few aspects, the structural homology of hormones and their receptors, the mode of action (including signaling pathways), presence of additional agents of ILPs signaling (e.g., insulin receptor substrate (IRS)), and the physiological role of ILPs (Table 1) [26,30,31,32]. In mammals, insulin is produced by β-cells of pancreatic islets, and in insects ILPs are mainly produced by brain insulin-like peptides and by cells in lateral organs like body fat or the gut [30]. Nevertheless, it is suggested that IPCs in vertebrates and invertebrates may be derived from a common ancestry, despite the great differences in their anatomical localization, because of similar developmental programs involved in their specification [33].

The common feature of insect ILPs is a conserved domain organization of their precursors, consisting of a signal peptide, with a B-chain, C-peptide, and A-chain, like the vertebrate ILP family. Mature peptides are heterodimeric and consist of the A- and B-chains linked by disulfide bonds, such as in vertebrate insulin or relaxins. They are formed by cleavage of signal peptide and removing C-peptide in the next step. The second group—the IGF-like peptides (e.g., Bommo-IGF-like peptide) holds the C-peptide, thus, they are single-chain peptides with internal disulfide bonds like vertebrate IGFs. The representatives of the third group, Dromo-ILP7-like peptides, are characterized by an unusually conserved sequence shared by several insect species [29,34]. The homology between amino acids sequence and 3D structure of mammalian insulin and insect ILPs is so high that Drome-ILP5 is able to bind and activate human insulin receptors (IR) and decreases glucose level in rats [35]. On the other hand, human insulin [36] and IGF1 [37] activate IR in mosquitoes *Anopheles stephensi*, porcine insulin activates IR in *Rhodniusprolixus* [38], and the *D. melanogaster* IR also binds mammalian insulin with reasonably high affinity [39].

Insulin biosynthesis in β-cells is regulated both at transcriptional and translational levels. For example, PAX6 transcription factor, a member of the PAX multigene family, is essential for the normal expression of insulin in β-cells [40]. Interestingly, it was shown, that *Drosophila* homolog of PAX6, an Eyeless (Ey) factor, directly promotes *dilp5* expression in neuronal insulin producing cells (nIPCs) in a nutrient-dependent manner [29]. There is another similarity between transcription factors included in insulin gene expression, the Dachshund (Dac) factor, which is a highly conserved nuclear protein—a critical transcription factor that specifically regulates *dilp5* expression by interaction with Ey factor. In mammalian β-cells, Dach1/2 (mammalian homolog of Dac) is responsible for proper interaction of PAX6 with insulin gene promoter in cultured mammalian β-cells [41]. Moreover, Dach1/2 is crucial for the development of β islet cells [42], and Dac factor also undergoes expression in insect nIPCs during the development stage [41], which suggests its role in proper development of those cells. It was also demonstrated that Dach1 is increased in the livers of obese mice and humans defecting insulin signaling, and thus promotes hyperglycemia and hyperinsulinemia state [43].

The most important physiological event that stimulates insulin gene transcription and mRNA translation is glucose metabolism. Moreover, glucose is essential for insulin mRNA stability, but the intracellular cAMP levels are also crucial for that process [44]. When rat islets were exposed to 25 mM glucose for 1 h, the intracellular proinsulin levels increased significantly. Similar situation occurs in insects, in which glucose is a widespread nutritional signal increasing ILPs level in hemolymph [45]. Glucose regulation of insulin release is mediated by metabolic signals and occurs in the following way. Pancreatic β-cells express GLUT2 glucose transporters, which rapidly uptake glucose into cells. At low glucose levels little substrate is phosphorylated by various hexokinase isoforms. The low expression of hexokinase results in a low glycolytic flux and low ATP/ADP levels. At high glucose levels, β-cells phosphorylate glucose *via* high-affinity glucokinase that increase in the ATP/ADP ratio. This leads to a proportional increase in overall glycolytic flux and mitochondrial glucose oxidation. That connection seems to be crucial for the next step of the signal generation in β-cells. High level of synthesized ATP by mitochondria is a trigger for ATP-sensitive K^+^ (K_ATP_) channels. Opening of the channels leads to membrane depolarization which opens L-type voltage-dependent calcium channels and finally induces exocytosis of insulin [46,47]. It was evidenced that insect IPCs respond to glucose and trehalose level in hemolymph, and their levels are sensed by sensors comprising, as in mammalian pancreatic cells, glucose transporters, K_ATP_ channels, voltage sensitive Ca^2+^ channels, and membrane depolarization [7,48].

The synthesis and secretion of insulin is also regulated by numerous hormonal factors. β-cells, for example, express cholecystokinin receptor 1 (CCK_1_ receptor). Its activation by CCK induces increased secretion of insulin during low glucose level [49]. It was also shown that CCK or its derivatives stimulate insulin secretion in both mice and patients with type 2 diabetes [50]. Moreover, low constitutive expression of CCK in β-cells is needed for keeping them in good condition. CCK probably prevents them from apoptosis, thus, allows them to maintain a proper number of β-cells in pancreas [49]. Insect IPCs also co-express sulfakinine (SK), a homologue of mammalian CCK [51]. It was shown that knockdown of either *sk* or *ilp*s affects the mRNA level of each other in *Drosophila* flies, suggesting that there is a cross talk between these two signaling pathways [51]. Additionally, recently, it was shown that SKs influence the ILPs level in the hemolymph of the *Tenebrio molitor* beetle [52]. Moreover, it was evidenced that ILP signaling regulates food intake via crosstalk with sulfakinin in the red flour beetle, *T. castaneum.* Silencing of the *InR* decreased the food intake leading to a decrease of weight gain and lowered the sulfakinin gene expression. However, double silencing of *sk* and *InR* eliminated the inhibitory effect on food intake as induced by silencing of *InR* [53].

Another neuropeptide which interplays with insulin in regulation of metabolism is substance P (SP) belonging to the tachykinin neuropeptide family. For example, this neuropeptide has a stimulative effect on insulin secretion from isolated rat islets [54] and decreases insulin level in blood in mice with diet-induced obesity [55]. Expression of receptors for TRPs in IPCs was confirmed also in *D. melanogaster*. TRPs knockdown significantly affected the mRNA levels of *dilp2* and *dilp3* in the brains of fed and starved flies [56]. In insects, ILP signaling also shows crosstalk with other neuropeptides: short neuropeptides F (sNPF) and allatostatins (Ast). This interplaying is described in further sections.

Insulin and ILP secretion are also regulated by non-peptide neurotransmitters and hormones. Acetylcholine released by parasympathetic nerve endings in the pancreas causes a potentiation of insulin release when glucose is present at concentrations greater than 7 mM. Acetylcholine binds to the muscarinic acetylcholine receptor M_3_ on pancreatic β-cells [57]. The binding has two effects: an increase in permeability of the cell membrane to Na^+^, and the activation of phospholipase C β-1 through a heterotrimeric G protein. Finally, an increased calcium level in cytosol is observed, which stimulates the exocytosis of insulin granules [57]. Chowański, et al. [58] showed that agonists of muscarinic cholinergic receptors applied exogenously increased levels of ILPs circulating in hemolymph of *T. molitor* larvae, whereas application of muscarinic receptors antagonist evoked opposite effects.

The ILP signaling pathway is initiated by the binding of ILPs to IRs. IRs are part of the tyrosine kinase (RTK) receptor family, which contains enzymatic tyrosine kinase domains within their cytoplasmic part [59]. In mammals, there are three ILP receptors: insulin receptor, IGF1 receptor, and IGF2 receptor. Each binds its ligand with 100–1000-fold higher affinity than for the other ILPs. Nevertheless, the IGF1 receptor and the insulin receptor can form functional hybrids that have similar affinities for insulin and IGF1. The IGF2 receptor, as it is distinct from the others, belongs to the G protein coupled receptors (GPCRs). The other receptor type for mammalian ILPs is a leucine-rich repeat-containing G-protein coupled receptor (Lgr) which binds relaxin with high affinity [25]. In insects, one or two IRs belonging to the tyrosine kinase family were identified. Moreover, recently in *D. melanogaster* the presence of relaxin-like receptors (Lgr3) was confirmed [60,61]. As was mentioned before, insulin is able to activate insect IRs as well as insect ILPs, e.g., Drome-ILP5 activates human insulin receptors.

In mammalian cells, the RTKs for insulin and IGFs potentially can activate two signaling pathways that affect cell metabolism and growth. One of them is activated when a ligand binds to the extracellular α-subunits of IRs, and then the kinase regions of membrane-spanning β-subunits undergo extensive tyrosine phosphorylation [32]. As a result of phosphorylation, receptors interact with adaptor proteins and insulin receptor substrates (IRS). These proteins then bind and activate either a growth factor receptor-bound protein (GRB2) or a phosphatidylinositol-3-kinase (PI3K), each of which initiates a distinct pathway [32]. Thus, IR stimulation typically activates two main intracellular pathways: the PI3K/Akt/FOXO (phosphatidylinositol-3 kinase/protein kinase B/Fork head box transcription factor) cascade and the Ras-MAPK pathway (mitogen-activated protein kinase) [38]. The signaling pathway occurring in insect cells after IRs activations has many common points with those described for mammalian cells. Ligand binding to IRs is a trigger activating receptor autophosphorylation and then leading to further phosphorylation of a range of downstream targets including CHICO, a major IRS for the *D. melanogaster* IR. Phosphorylated CHICO binds to PI3K and induces its activation and production of (3,4,5)-trisphosphate (PIP3). Accumulation of PIP3 leads to activation of the protein kinases dPDK1 (*D. melanogaster* phosphoinositide-dependent kinase-1) and dAkt (protein kinase B) which finally activates dFOXO [31]. dFOXO—an insect homolog of mammalian FOXO is activated inter alia during amino acid starvation which is crucial for survival under nutrient-restricted conditions [62] and overexpression of dFOXO closely mimics the phenotypic effects of starvation, suggesting a role for dFOXO in the response to nutritional adversity [63]. The Ras/MAPK cascade can be activated, besides phosphorylation of CHICO, by phosphorylation of other IRS such as Shc [64].

The mammalian and insect ILP signaling pathways have more common points. For example, in insects, the presence of homologs of mammalian insulin-degrading enzyme (IDE) were confirmed. When the IDE was knocked down in *D. melanogaster* IPCs, an increased body weight and fecundity, decreased circulating sugar levels, and reduced lifespan were noticed [65]. In addition, in mammals, IDE is important for insulin and glucose homeostasis. The enzyme activity and protein level of IDE were increased in the liver of diet-induced obese mice [66], while, in mice with lost function of IDE in liver, hyperinsulinemia and insulin resistance were observed [67].

It should be mentioned that there is homology between mammalian insulin-like growth factor binding proteins (IGFBP) and acid labile subunit (ALS) and insect imaginal-morphogenesis protein Late-2 (Imp L-2) and neuroparsins and dALS. IGFBP and ALS take part in regulation of IGFs level in serum. They have abilities to bind the IGFs and form a ternary complex. In this way, they control the level of free active form IGFs in serum. There is evidence that the IGF-IGFBP-ALS system has an important pathophysiological role across the spectrum of obesity, insulin resistance and type 2 diabetes mellitus [68]. Interestingly, RNAi-mediated silencing of dALS results in increased body mass, reduced hemolymph sugar level, and increased lipid storage in *D. melanogaster* [69] whereas injection of neuropasin-A increased hemolymph trehalose level and lipid levels and body weight in *L. migratoria* [70].

There are many studies showings that nutrient metabolism, body weight, growth, and other physiological processes are controlled in similar ways by ILPs in mammals and insects, demonstrating that insects can be a good model for studies concerning ILPs in the development of obesity. In previous subsections, we described examples of homology between ILP systems in both mentioned groups of animals. So now is time to provide a few examples on a physiological level. Ablation of brain IPCs causes increased hemolymph sugar levels, suggesting a diabetic-like phenotype in larvae of *D. melanogaster* and in adult flies, the increased storage of lipids and carbohydrate, increased resistant against starvation and oxidative stress and the extending life span were observed. However, when the *dilp2* was overexpressed, reduction in hemolymph sugar level and body weight were noticed [26]. Glucose stimulates secretion of Bommo-ILPs into hemolymph, and they decrease trehalose level in hemolymph and increases trehalase activity in muscle and midgut, which increases trehalose uptake into cells, suggesting that Bommo-ILPs promote consumption of carbohydrates [71]. In the mosquito *A. aegypti*, Aedae-ILP3 reduces circulating sugars 6 h after injection, but it also elevated carbohydrate and lipid storage 24 h after injection. This resembles the action of insulin in mammals [72]. The physiological effects of ILPs in insects depend on diet, which was confirmed by Semaniuk, et al. [73]. For example, they showed compensatory feeding effects for carbohydrates depending on nutrient composition. For more examples see the reviews [32,33,37].

### 2.2. Adipokinetic Hormones

Besides ILPs, adipokinetic hormones are one of the most important insect neuropeptides involved in regulation of insect metabolism. They are a physiological counterpart of mammalian glucagon acting antagonistically to insulin/ILPs (Table 1) [11]. Under low blood sugar conditions, α-cells release glucagon, which triggers the breakdown of glycogen to free sugars. Glucagon is also a lipolytic hormone that regulates lipid concentrations in plasma [74], the similar function have insect AKHs [75]. Structurally, AKH is octa- or decapeptides but other forms are also reported [76], so they are definitely shorter than glucagon which contain 29 amino acid residues. Nevertheless, they share some similarity in amino acid sequence, Thr-Phe_(5–6)_X-X-Asp and Thr-Phe_(3–4)_X-X-Asp for glucagon and AKH, respectively [74]. The neurosecretory cells synthesized, storing and secreting AKH are mainly localized in *corpora cardiaca* and are functional analogues of vertebrate pancreatic α-cells [33]. As mammalian and insect IPCs and pancreatic α-cells have a neurodermal origin [33], the AKH producing cells (APCs) have mesodermal origin [77]. Nevertheless, AKH is also produced in other tissues/organs such as ovaries, midgut, fat body, accessory glands, and muscle tissues [75].

In mammals, the main triggers for glucagon release are low blood glucose level, protein-rich meals and adrenaline [78]. In insects, sugars are known to positively stimulate APCs to release AKH in larvae [79]. Moreover, gustatory and odor perception and water sensing-gustation are involved in promotion of AKHs secretion by APCs [74]. *akh* gene transcription is negatively regulated by AKH signaling. In addition, the AMP-activated protein kinase (AMPK) seems to be a crucial factor that promotes AKH secretion [80]. It was also shown that expression of K^+^ ATP-dependent channels which take part in triggering of glucagon secretion in α-cells [81] are presented also in APCs [7].

The AKHs released to hemolymph act on cells by activation adipokinetic hormone receptors (AKHR), a rhodopsin-like G protein-coupled receptor, which is related to the vertebrate gonadotropin-releasing hormone receptors [82] which was firstly identified from *D. melanogaster* and the silkworm *B. mori* [75]. The main place of *akhr* expression is fat body, but AKHR was also localized in other tissues such as the midgut, muscles, brain, and reproductive system [83,84]. The action of AKH on target cells and lipid mobilization by AKHR activation might occur in two different ways. In the first one, the activated AKHR switches on the phospholipase C (PLC) signaling pathway. PLC converts membrane phosphatidylinositol 4,5-diphosphate (PIP_2_) into inositol-1,4,5-trisphosphate (IP_3_) and diacylglycerol (DAG). The released IP_3_ diffuses through cytoplasm and activates the IP_3_ receptor (IP_3_R) in the endoplasmic reticulum. That cause the release of Ca^2+^ ions into cytosol which activate the machinery responsible for lipid mobilization and glucagon hydrolysis into glucose, but the mechanism is not fully known [85,86]. The alternative AKH signaling pathways includes activity of adenylate cyclase and mediates a rapid increase of the cAMP leading to activation of cAMP-dependent protein kinase (PKA). Activation of PKA promotes the phosphorylation of downstream elements, such as the triglyceride lipase (TGL) and Perilipin 1/Lipid Storage Droplet-1 Protein (PLIN1/LSD1) [87,88,89]. PLIN1 phosphorylation has been shown to increase the accessibility of lipid droplets for TGL, thereby allowing lipid mobilization in *Drosophila* [90]. The lipolytic properties of glucagon and participation of perilipin in that process were shown also in mammals: in rats, rabbits’ adipocytes, and human adipocytes in vitro [91]. The active form of perilipin affects the AMP-activated protein kinase (AMPK) family. It inhibits the salt-inducible kinase 3 (SIK3) resulting in activation of the transcription factor forkhead box class O (FOXO) which promotes expression of *bmm* gene encoding Brummer (bmm) lipase (homolog of mammalian adipose triglyceride lipase (ATGL)) [92,93]. Moreover, a serine/threonine kinase known as liver kinase B1 (LKB1) also plays an important role in governing lipid metabolism by activating SIK3 in a kinase activity-dependent manner [89,94].

RNAi-mediated knockdown of AKHR in the fat bod reduces diacylglycerol (DAG) levels leading to triacylglycerols (TAG) accumulation in the fat body in the kissing bug *R. prolixus* [95], cricket *Gryllus bimaculatus* [96], and fruit fly *Bactrocera dorsalis* [97]. Dual knockout of the AKHR and bmm genes in *Drosophila* flies caused flies to be obese and starvation-intolerant [75,98]. Similarly, null mutation in AKH results in obese *Drosophila* adults, whereas its over-expression leads to a dramatic reduction of lipid stores. Gáliková, et al. [99] confirmed an orexigenic activity of AKHs. They also showed that AKHs affects expression of orexigenic factors such as CCHamide-2 and neuropeptide F as well as metabolic-related genes like corazonin, limostatin, and ILPs [99]. In the case of NPF the opposite interaction was confirmed, when this peptide regulates lipid metabolism through AKHs signaling [100]. Interactions in metabolism regulation between glucagon and neuropeptide Y (NPY) were also shown in mammals in experiments conducted on pigs [101] and on isolated mice pancreatic islets [102]. The cellular calcium homeostasis is crucial for both signalizations occurring by glucagon and glucagon receptors as well as by AKH and AKHR. For example, impaired of sarco/endoplasmic reticulum calcium-ATPase (SERCA) activity leads to reduced fat storage in adipose tissue in *Drosophila* [103] whereas loss of IP_3_R leads to obesity in adults individual of that species [104]. For more examples see the review Toprak [75].

AKHs affect not only lipid metabolism but also carbohydrate metabolism. The mobilization of insect fat body glycogen by AKHR activation occurs through activation of glycogen phosphorylase. It was shown that the glycogen phosphorylase activity depends on Ca^2+^ influx of extracellular calcium into the fat body cells and intracellular level of cAMP [105]. Enhanced response of *Drosophila* AKH in the fat body is essential for hyperglycemia associated with a chronic high-sugar diet [106]. These authors showed that the activin type I receptor Baboon (Babo) autonomously increases AKH signaling without affecting ILP signaling in the fat body via, at least, increase of AkhR expression. Moreover, activin-β, a Babo receptor ligand whose secretion by midgut cells is stimulated by a chronic high-sugar diet, promotes AKH action in the fat body, leading to hyperglycemia. Importantly, activin signaling in mouse primary hepatocytes also increases glucagon response and glucagon-induced glucose production [106].

Despite AKHs not being structural homologs of mammalian glucagon, they show evident similarities in their action to glucagon activity. Thus, the studies concerning the mechanism of AKHs action in insects might be a source of knowledge that can help better understand the role of glucagon signalization disturbance in development of obesity and other metabolic disorders.

### 2.3. Corazonin

A neuropeptide named corazonin (Crz) is an 11 amino acid peptide which is found to be homologous to human gonadotropin-releasing hormone (GnRH) (Table 1) [75,107]. Corazonin is a common peptide through arthropods, but its absence was noticed among Coleoptera [107]. Research showed that receptor for corazonin is highly selective and for receptor–ligand action the C-terminus of peptide is necessary; however, a structural similarity between between Crz and *Drosophila* AKH receptors was found [7,108,109]. As Kubrak, et al. [110] suggest, the common signaling system for Crz, AKH, and GnRH might be an effect of one ancient and conserved ancestral evolution [109,110].

Corazonin is synthesized inter alia in brains lateral neuroendocrine cells as well as in interneurons of the abdominal ganglia of ventral nerve cord [75,107]. It was found that receptor for Crz is expressed in embryos, larvae, pupae, and adult insects in which is mainly expressed in salivary glands and fat body tissue which is counterpart of human adipose tissue/liver [108,110]. Function of corazonin is wide but mainly concerns processes related with initiation of ecdysis, melanisation, copulation, vitellogenin gene expression, and social behavior while its first described cardioacceleratory action is strict to a few cockroach species (e.g., *P. americana*) [7,75,107,108,111,112]. Few researchers suggest that function of corazonin might also be connected with feeding- and stress-induced starvation/nutritional stress and might lead to regained metabolic homeostasis [108,113]. This suggestion is supported by results obtained from *Drosophila* with ablated neuroendocrine cells which produce corazonin, in which trehalose level was 15% reduced in relation to control insects [114,115]. It was explained by the relation of Crz which probably affects APCs, which is possible thanks to close localization of corazonin-immunoreactive axons and *corpora allata* in which AKH is produced [114,116]. In addition, knockdown of *Drosophila CrzR* genes in fat body tissue as well as salivary glands caused reduction of food intake and increased resistance to starvation [108]. This knockdown at the same time affects expression of genes encoding Drome-ILPs in fat body tissue and decreases TAG level [108]. However, the ablation of corazonin expressed neurons localized in the brain caused increased TAG as well as circulating and stored glucose levels [75,108]. It was also noticed that corazonin might interact with ILP signaling pathways and in that way act as an indirect stimulator of lipolysis [75]. Studies conducted by Kubrak indicated also that *Crz* knockdown affects glycogen levels by increasing it 1.5 times [108]. Those differences were even more marked in insects after starvation (approx. three times) [108]. In addition, Crz interacts through neuronal pathways with AKH producing cells, but might also regulate trehalose levels acting as a hormone on fat body cells [89,114]. These observations are supported with ones carried on adult *Drosophila* flies with activated corazonin producing neurons which leads to increased food uptake [115,117]. There may also exist feedback from brain and *corpora cardiaca*, which is supported with results which showed that knockdown of corazonin receptor alters peptides, like dilp3, dilp5 and Crz and AKH levels [108].

### 2.4. Hugin

Neuropeptide hugin is a product of the *hugin* gene, which in *Drosophila* potentially encodes several neuropeptides [118]. At least two of them, hugin γ and Drome-PK-2, share a common PRLa motif at C-terminus, characteristic to the insect pirokinin/pheromone biosynthesis activating neuropeptide (PK/PBANs) family [118,119]. One of the *hugin* expression products is a homolog of mammalian neuromedin U8 (NMU8), which is responsible for coordination of feeding behavior among rodents (Table 1) [10,118,120,121]. Expression of genes encoding hugin takes place in a small group of 20 cells of the subesophageal ganglion as a part of the central nervous system (CNS), which are engaged in modulating feeding behavior in response to gustatory/nutrient signals [118,120]. In turn, NMU and NMU-expressing cells in mammals are localized in similar structures of nervous system, the arcuate nucleus of the hypothalamus, the pituitary, the *medulla oblongata* and the spinal cord yet the redistribution is more complex [122]. Hückesfeld, et al. [123] showed that hugin may be engaged in avoidance of bitter taste, which is often correlated with toxic substances, unless hugin neurons are not required for salt or sweet taste recognition. Research conducted by Melcher and Pankratz [120] indicates also that expression of *hugin* takes place in two additional structures strictly involved in processes correlated with food intake and metabolism: the ring gland as a neuroendocrine organ which controls metabolism and growth and the pharyngeal muscles which are engaged in food pumping [120]. Research showed that blocking transmission by synapses by tetanus toxins causes changes in initiation of food intake which depends on the previous nutritional state [120]. It is possible that the functioning of hugin in a neural circuit which modulates feeding behavior depends on chemo/taste-dependences [120]. It was shown that flies in two strains, *klu*- and *ppl*-mutants, in which expression of *hugin* was upregulated were wandering around (locomotion promotion) and not taking food [120,122]. A similar situation occurs in the case of homologous hugin—NMU [122]. In mammals, neuromedin U acts on regulation of energy homeostasis as well as on feeding behavior, by inhibiting food intake, but also promotes locomotion [122,124]. Decreased food intake caused activation of hugin neurons which at the end leads to a decreased activity of the pharyngeal pump [124]. In the case of insects with downregulated *hugin*, a lower level of hug neuropeptides correlates with hyperactive food searching and intake [120]. Melcher, after in situ hybridization, correlated obtained results with those previously mentioned and suggested that hug neurons may be directly engaged into regulation of first phase of feeding, which will explain food intake behavior in flies with decreased level of hugin [120]. Given these similarities, it was proposed that hugin is homologous to central NMU [122].

### 2.5. Unpaired-1 and Unpaired-2

Among insect groups are two analogues of leptin, mammalian hormone, which is also called “satiety hormone” thanks to its well described function in weight regulation, might be distinguished [125,126,127]. They are called unpaired-1 (upd1) and unpaired-2 (upd2) [5,125]. Both are *Drosophila* cytokines in which amino acid sequences suggest the presence of structural similarity to vertebrate leptins (Table 1) [5,128,129]. The first one is produced in the flies’ brains, while body fat is the only tissue that secrete upd2 as an active protein [5,125,130]. As shown in the research disturbances in upd1 and upd2 might lead to emergence of obesity signs but their occurrence proceeds differently [5,125]. Upd1 acts *via* domeless receptors whose presence was confirmed on neuropeptide F positive neurons [125,126]. Disruptions in upd1 activity/protein level causes intensified attraction to food odor cues as well as increased food intake which causes weight gain [128,131,132]. Disruptions in upd1 activity/level probably increased the activity of NPF which is fed stimuli, and in that indirect way causes obesity-related effects [125,126]. Additionally, it was shown that human leptin might activate flies’ Domeless receptors for upd1 in a manner characteristic for leptin receptors [5,133,134].

Another analogue of mammalian leptin is upd2, secreted by fat body in well-being flies, just like leptin released from adipocytes. The mechanism of action is different from upd1. Similarities between vertebrate leptin activity and *Drosophila upd2* are visible when flies are exposed to high fat diets [5]. Under conditions of nutritional abundance, upd2 works *via* JAK/STAT signaling pathway, which leads to increased secretion of Drome-ILPs from brain IPCs into hemolymph. This results in increased fat storage and finally growth [5]. Studies showed that in insects with mutation in *upd2* the appropriate loop controlling feeding might be rescued by leptin. Those studies were conducted on flies in which the expression of leptin was assured by transgenic gene [5].

Studies conducted on flies showed that both fat body- and brain-derived upd may act to control different aspects of weight regulation and growth [5,125]. Thanks to similarities in activity of upds and leptin, *Drosophila* with genetically induced obesity (e.g., upd1Δ or upd2Δ mutants) might be used as a good model for human obesity [5,125].

### 2.6. Allatostatin-A and C

In the search for new models for studying obesity, the homology observed between insect allatostatins, vertebrate somatostatin (SST), and galanin (GAL) [11,75] is of interest (Table 1).

Allatostatins (ASTs) are one of the largest family of insect neuropeptides [127]. They participate in regulation of many life processes, including regulation of juvenile hormone synthesis and modulation of contractile activity of heart and visceral muscles [128]. Moreover, ASTs are probably crucial for processing of optical information [129]. Current research indicates that ASTs are strongly involved in the regulation of insect feeding, nutrition, and post-feeding physiology [130,131]. Research conducted by Aguilar, Maestro, Vilaplana, Pascual, Piulachs and Bellés [130] showed that AST, despite regulation of hindgut contractility, also activates α-amylase secretion in midgut of the cockroach *Blattella germanica.* Moreover, ASTs application led to inhibition of food consumption by tested cockroaches [130]. Further research concerning regulation of food intake by ASTs strongly supports these results. For example, genetically based manipulations of neuronal activity performed by Hergarden, et al. [132] showed that neurons expressing the AST A modulate feeding behavior of adult *D. melanogaster.* Especially the changes were observed in the case of responsiveness of flies to a sugar diet. However, these changes are not directly related to insect metabolism, which suggests that activation of AST A neurons are a consequence not a cause of metabolic changes that induce the state of satiety [132]. In addition, research by Hentze, et al. [133] present that AST A and Dar-2 (receptor for AST A) are modulated differently by carbohydrate-rich and protein-rich diets in fruit fly *D. melanogaster*. Generally, AST signaling probably regulates feeding choices/decisions between different types of nutrients, which is crucial for balance of food intake and metabolic needs [133]. Interestingly, flies lacking AST A increased intake of sugar which led to increasing lipid storage droplets in fat bodies [133]. Not only AST A, plays a role in regulation of food intake. Research by Kh and Keshan [134] also suggests that AST C may be crucial for insect feeding behavior. In this research, the authors showed that the gene encoding AST C is expressed in the central nervous system of *B. mori* during starvation [134].

Allatostatin also may indirectly influence insect food intake and regulation of energy homeostasis. This is related to the fact that ASTs action is closely associated with AKH. The research conducted by Hentze, Carlsson, Kondo, Nässel and Rewitz [133] proved that transcript characteristic for the gene which encodes Dar-2 receptor was found in IPCs and APCs. Moreover, silencing of *Dar-2* causes changes in the expression level of genes connected with ILP and AKH signaling. Moreover, *Drosophila* flies lacking AST A accumulate high lipid levels [133]. That suggests that AST A is crucial for maintaining balance between these two neuropeptide families in response to changing ratios of dietary sugar and protein. Similarly, to somatostatin which inhibits both insulin and glucagon secretion in mammals. Exemplary studies using somatostatin receptor-deficient mice revealed changes in both basal and stimulated insulin secretion [135].

Presented examples of regulatory role of ASTs clearly indicate their structural and functional homology with vertebrate GAL and SST, hormones which are closely connected with obesity. Galanin is widespread in the vertebrate nervous system, including hypothalamus, which mainly participates in regulation of feeding behavior [136]. Generally, this hormone regulates many events related to food intake, starting from stimulation of food intake and weight control of animals and ending to regulation of intestinal motility [137]. All of these prove that between GAL and AST exists a strong functional homology. This assumption supports the fact that GAL signaling, similar to AST, helps adapt food intake and metabolism to changes in animals’ diet [136]. Another example which presents close functional homology between AST and GAL, is the close relationship between GAL-signaling and insulin-signaling [138]. Despite functional homology between insect ASTs and vertebrate GAL, between these two hormone families also exist some structural similarities, because Dar-2 is considered as a homologue of GAL receptor [76,117].

Regardless of functional and structural homology with GAL, some resemblances between AST and vertebrate SST also exist [139]. This homology is mainly associated with the fact that AST, similar to somatostatin, can be produced not only in CNS, but also in other tissue, such as midgut and possess inhibitory properties [11,127]. In addition, the presence of a disulfide bridge between C3-C14 and the close relation between somatostatin/galanin/opioid receptor and AST receptors indicate that they share a common evolutionary origin [11].

### 2.7. Sulfakinins

Cholecystokinin is a regulatory peptide with a conserved C-terminal amino-acid sequence (-XMXFa). Its family embraces not only CCK but also gastrin (G) in mammals, skin peptides isolated from frogs (caerulein and phyllocaerulein) and peptide cionin in protochordata [140]. In invertebrates the CCK/G family consists mainly of sulfakinins with C-terminal -HMRFa and sulfated tyrosine residues -XY(SO_3_)HGHMRFa. Thus, sufakinin resembles mammalian CCK/G in structure of the molecule [140,141]. Sulfakinins have been first identified in *Leucophaea maderae* based on their myostimulatory activity [141] and nowadays, they are present in various insect species which proves their high conservation across insects and importance as signaling molecules [52,142]. The precursor sequence in all tested insect species contains two peptides from which both sulfated and nonsulfated forms of peptide are present and physiologically important [143]. This is also observed in CCK/G peptide family in mammals [140].

Apart from structural homology, SKs seem to have similar physiological functions as CCK in mammals (Table 1). In insects, SKs are suggested to be a satiety factor and regulator of gut physiology. Injections of this peptides were shown to inhibit food intake in *Schistocerca gregaria*, *B. germanica*, *Phormia regina*, and *R. prolixus* [144] whereas silencing of SK gene in *G. bimaculatus* and *T. castaneum* stimulate food intake [145,146]. Furthermore, SKs were shown to affect the contractions of gut contractions however the results were contrary. They stimulate contractions in *L. maderae*, *Zophobas atratus*, *R. prolixus,* and *L. migratoria* [144,147] whereas in flies *P. regina* and *Calliphora vomitoria* no myostimulatory effect was observed [144].

The mechanism of action of sulfakinins, as almost all signaling molecules, is based on interaction with its receptors. In insects sulfakinin receptors (SKRs) were described in several insects including *R. prolixus*, *P. americana*, *T. castaneum*, *D. melanogaster,* and *T. molitor* [52,142]. In all species two receptors SKR1 and SKR2 were found and described as seven transmembrane receptors with N-terminal extracellular and C-terminal tail coupled with regulatory G protein. Detailed amino acid analysis showed that they can be described as rhodopsin-like family of GPCRs and display quite a high degree of homology (over 30%) with their human homologs—cholecystokinin receptors [52,142]. Furthermore, in *R. prolixus*, it was shown that activation of the receptors triggers intracellular Ca^2+^ pathway via Gαq as in mammalian and nematode CCKRs [142]. Distribution of SKRs is also quite similar to that of mammalian CCKRs and in most insects is the highest in the central nervous system and alimentary tract which resemble the physiological role of these peptides in gut physiology related to food intake [52]. In insects tissue specific distribution of both SKRs again comply with the one for CCKR in mammals [146]. For instance, in humans, CCKR1 is involved in inducing satiety, slowing down gastrointestinal motility, and stimulating secretion of pepsinogen, while activation of CCKR2 can stimulate nociception, memory and learning processes, panic and anxiety, endocrine pancreas secretion, gastric acid secretion, and gastric mucosa growth. In *T. molitor*, it was shown that SKR2 is much more abundant in the CNS than SKR1 which is probably in agreement with the situation in mammals [52].

The metabolic effects of SKs in insects is the next similarity between SK signaling and CCK/G signaling. It is widely known that CCK influences the metabolic/energetic status of the organism in mammals [148]. In insects SKs were shown to influence various metabolic pathways. Injections of sulfated and nonsulfated SKs increase lipids and proteins in the fat body and total carbohydrates in the hemolymph of *T. molitor* beetle, simultaneously decrease the glucose concentration [143]. Recent studies showed that T. *molitor* SKs are also involved in fatty acid metabolism [149]. Considering all described similarities between SKs and CCKs, extensive research on SKs role as peptides active in brain–gut axis in the same way as CCK in mammals may lead to development of active substance which may contribute to discovery of anti-obesity agent.

### 2.8. Short Neuropeptides F

Another important signaling molecules which regulate feeding and energy homeostasis in insects are sNPF. According to the nomenclature, they were firstly connected with another group of peptides with similar C-terminus neuropeptides F (NPF) described later in this article. It was confirmed that both groups have a common ancestor but the sNPF signaling system is found only in protostomes and probably in echinoderms [150]. Nevertheless, sNPF receptor show similarity to prolactin releasing peptide receptor (PrPR) which is known to be involved in regulation of feeding behavior in mammals (Table 1) [151]. This was also confirmed by phylogenomic analysis [152]. First sNPF (designated as head peptide), was identified in the midgut of *P. americana* and later in the brain of the beetle *Leptinotarsa decemlineata* [151]. Since that time, a huge number of sNPFs sequences have been identified in various insects, i.e., fly *D. melanogaster*, mosquito *A. aegypti*, moth *B. mori*, or various beetles [151]. Although sNPF has several described physiological functions, it seems to be mainly involved in the regulation of feeding; however, the effects are probably species specific [126]. In *D. melanogaster* sNPFs increase food intake and interplay with ILP signaling pathways [28]. In *Drosophila*, some dorsal lateral peptidergic neurons terminating on IPCs express sNPF whereas IPCs express sNPF receptor 1 (sNPFR1) and corazonin receptor (Crz-R) [153]. Knockdown of *snpf* in those neurons decreases the levels of mRNA for *dilp2* and *dilp5* in the brain and prolongs survival in starved flies and changes the carbohydrate and lipid metabolism [115,153].

Contrary to flies in mosquito *A. aegypti* and locust *S. gregaria* sNPF was shown to be a satiety factor. On the other hand, in the cockroach *P. americana* again sNPFs level was elevated in starved animals, as in *Apis mellifera* [126]. Nevertheless, in diapausing *L*. *decemlineata* beetles sNPFs were not present, which suggest that it is important in actively feeding insects.

sNPF receptor (sNPFR) belongs to the subfamily A of rhodopsin-like GPCRs. The in silico analysis showed that based on sequence similarities, receptors for the sNPFs cluster together with the receptor for prolactin-releasing peptide [154]. For example, sequence similarity between *T. molitor* sNPFR and human PrRP is about 40% [151]. This suggests that sNPF may have evolved into the prolactin-releasing peptide signaling system, that also regulates feeding and has been suggested to be orthologous to sNPF [126]. PrRP is a member of RFamide peptide superfamily and in mammals its role, despite the name, is no longer associated with release of prolactin. It has been proven that this peptide in vertebrates is involved in the regulation of feeding [155], indicating the structural and functional similarities of sNPF and PrRP between insects and mammals. Moreover, PrRP analogues and PrRP receptors are considered in anti-obesity therapy [155]. This is an argument to study insects sNPFs biology in terms of obesity.

### 2.9. Neuropeptide F

Neuropeptide Y in mammals is a member of a large neuropeptide family which also embraces peptide YY (PYY) and pancreatic polypeptide (PP) [156]. It is a 36 amino acid pleiotropic signaling molecule widely present in the central and peripheral nervous system with a major role in feeding regulation and maintaining energy homeostasis [156]. In invertebrates, NPY-like peptides were discovered in a variety of species including flatworms, mollusks, and insects. Based on its C-terminal sequence that ends with a phenylalanine (F) instead of a tyrosine (Y), these peptides were named neuropeptides F (NPFs) instead of neuropeptide Y (Table 1) [157]. After issues with nomenclature, due to C-terminal similarity with sNPF, all invertebrate NPFs display RXRFa C-terminal sequence and 28 to 40 amino acid length [126]. For this reason, they are often called long/true NPFs.

In general, in insects NPF was shown to be orexigenic similarly to mammalian NPY. Experiments with gene overexpression in *D. melanogaster* or injections of NPFs to *S. gregaria* stimulate food intake [126]. However, this feeding response integrates food attractiveness and hunger state [158]. NPFs thus regulate food choice behavior. In satiated flies it was shown that NPF expression is inhibited, and animals prefer high quality food instead of lower quality or noxious [158].

In the regulation of metabolic homeostasis on the level of whole body NPFs also display similarities with mammalian NPY. They interplay with ILPs and Upd1 in *D. melanogaster* which is a homolog of mammalian leptin as described above. Upd-1 receptor is expressed in NPF neurons in *D. melanogaster* brain and smell activated appetite is perturbed in flies lacking *Upd-1* gene. In turn, flies do not react on satiety, consume food as starved flies, and become obese [125]. This makes the NPF/Upd1 system a perfect target in studying obesity grounds.

NPF receptors (NPFR) belong, as almost all insect neuropeptide receptors, to GPCRs. In *D. melanogaster* cloning and functional expression of two NPFRs revealed that they could be activated by mammalian NPY and peptide YY [126]. Later, *D. melanogaster* NPF was shown to dose-dependently activate NPFR when expressed in Chinese hamster ovary (CHO) cells [126]. Such a cross-activity might give the opportunity to use this system in studying the interspecies role of NPFs.

### 2.10. Tachykinin-Related Peptides

Tachykinin-related peptides are a large family of insect neuropeptides, which they name and possess structural similarity to vertebrate TKs. The structural homology is so high that it causes activation of insect TRP receptors by substance P (SP, one of the mammalian TKs) [159]. Between insect TRPs and vertebrate TKs exist not only structural similarities, much research concerning this issue showed a wide spectrum of functional homology of these neuropeptides (Table 1). For example, members of both families participate in the regulation of visceral muscle contractions, nociception, and immune system activity [11,160]. In addition, TKs and TRPs probably play a very important role in modulation of lipid metabolism [161,162].

In mammals, TK-signaling is linked with obesity for many years, especially associated with presence of SP and its receptor NK-1R (neurokinin-1 receptor) [163,164]. For example, Baroncelli, et al. [165] showed that obese children had elevated level of SP. In addition, presence of SP in intestines and the ventromedial areas of the brain, which participate in modulation of feeding behavior, may suggest regulatory role of SP in food intake [164,166]. This assumption was confirmed, for example by Karagiannides, et al. [167]. These authors showed that administration of SP antagonist which bind to NK-1R, reduced weight gain and fat accumulation and improved insulin sensitivity in obese mice [167]. In addition, research by Fu, Liu, Liu, Liu, Li, Wu and Liu [164] and Ramalho, et al. [168] suggest that SP might play an important in obesity and also for developing T2DM (type-2 diabetes mellitus) and asthma in obese patients, by increasing serum levels of IL-6, which is inextricably linked with tissue inflammation.

In addition, in insects TRPs participate in regulation of fat storage, by suppression of lipogenesis. Research by Song, Cheng, Hong, Sappe, Hu, Wei, Zhu, O’Connor, Pissios and Perrimon [106] on *D. melanogaster*, showed that TRPs repress of the lipogenic SREBP (sterol regulatory element-binding protein), membrane-bound transcription factors which control genes participating in regulation of cholesterol and FAs synthesis. This phenomenon was observed during fasting, because in this physiological state TRPs are released from neuroendocrine cells [161]. In addition, TRP deficiency flies lead to an increase in TAG levels in the midgut and fat body [161]. Moreover, current research suggests a close relationship of TRPs with AKH signaling. For example presence of TRPs lead to release of AKH from the retrocerebral complex of locust *L. migratoria* [169]. Moreover, *Akh Drosophila* mutants (knockdown of gene encodes AKH precursor) are characterized by elevated gene expression for TRPs [75,99]. All these results may suggest that suppression of TRP signaling may contribute to the AKH deficiency-triggered obesity [75,99].

### 2.11. CAPA-PVK Neuropeptides

The first identified member of insect CAPA-PVK neuropeptides was Manse-CAP2b1 (cardioacceleratory peptide 2b) isolated from the ventral nerve cord of the *Manduca sexta* moth [170]. Its name comes from the cardioacceleratory properties and stimulation of contraction of visceral muscles. However, most research suggests that these neurohormones are primarily involved in regulation of insect diuresis [171,172,173,174,175,176,177,178,179,180,181].

Research by Terhzaz, et al. [172] confirms that not only are hugins the NMU homologues, but also insect CAPA-PVK neuropeptides. In this research, authors present that vertebrate NMU is a putative functional homolog of Drome-CAPA-1 and -2. Structural and functional homology between these neuropeptides is confirmed. For example, CAPA-PVK neuropeptides and NMU participate in modulation of visceral muscle contractions [136,182,183]. Moreover, both neuropeptide families are involved in regulation of ion transport and participate in regulation of immune system activity [180,182,184].

As we mentioned previously, the current results showed that NMU reduced food intake and simultaneously led to an increased locomotor activity, which has a crucial role in regulation of glucose homeostasis in blood. In addition, mice with silenced NMU genes were characterized by hyperphagia, hyperglycemia, and lower locomotor activity, which led to obesity [182,183,185]. The research conducted on carp *Cyprinus carpio* confirmed this observation, because only in satiating individuals’ higher expression of gene encoding NMU precursor in brain and gut were observed [173]. For these reasons, NMU is considered as a potential compound which might be used in therapy of obesity [174].

Despite the previous mentioned role of CAPA-PVK in regulation of water homeostasis, current research showed that these neuropeptides, similar to NMU, can also participate in regulation food intake (Table 1) [175]. Research by Sangha, Nachman, Lange and Orchard [175] demonstrated that 2129-SP3[Φ3]wp-2, one of the CAPA-PVK analogue which is characterized by higher stability in insect hemolymph, induced the intake of a larger blood meal by *R. prolixus*. Interestingly, application of native Rhopr-CAPA-2 did not influence the size of blood meals. As the authors suggest, these results may indicate that RhoprCAPA-2 might not influence satiety but is more a signal to prevent additional feeding events [175].

### 2.12. Other Peptides

#### 2.12.1. RYamides

Other peptides which are considered as a food intake regulator were discovered 10 years ago as RYamide neuropeptides [176]. They were first connected due to some structural features with NPY but after phylogenetic analysis put to the separate luqin/RYamide neuropeptides family, which in mollusks was suggested to regulate various physiological processes including feeding and water homeostasis [177]. In insects the exact physiological role of these peptides is not known; however, there is some evidence that they might be involved in feeding and digestion. In flies RYa decreases feeding motivation [176] whereas expression in enteroendocrine cells in *B. mori* suggest the role in digestion [178]. RYamides secreted from the enteroendocrine cells of midgut might act as a neuromodulator directly on the hindgut to suppress its contraction, inducing a larval feeding state transition from the feeding- to the non-feeding mode [177]. Even though the luqin/RYamide signaling system is probably lost in vertebrates, its possible role in feeding regulation might be the target to study anti-obesity factors.

#### 2.12.2. CCHa-2

The next group of neuropeptides considered as brain–gut peptides in insects, thus with potent usage as an obesity target, is the CCHa family of peptides. They were first discovered in *B. mori* and were shown to possess conserved cysteines and amidated histidine at the C-terminus [179]. These peptides do not have the mammalian analogue but were shown to be involved in feeding regulation. Ren, et al. [180] showed that in a Capillary Feeding (CAFE) assay, *D. melanogaster* flies with disrupted *Ccha2* gene show a significantly reduced food intake. Furthermore, injection of CCHamide-2 into living blowflies *Phormia regina* resulted in an increased number of the proboscis extension reflexes when sucrose solutions were offered to these animals, which proves that CCHa-2 stimulates the feeding motivation in flies [181]. All above data conclude that CCHamide-2 is an orexigenic (stimulating appetite) peptide in *D. melanogaster*. Additional phylogenetic studies showed that CCHa receptors group together with mammalian BRS-3 orphan receptors [181] but thus far pharmacological assays showed no clear effects. Mice with knocked *BRS-3* showed metabolic defects and developed obesity so ligands of mammalian BRS-3 might be an inhibitor of food intake.

#### 2.12.3. SIFamides

SIFamides are the next group of neuropeptides involved in regulation of various physiological processes including feeding. They were first isolated from flesh fly *Neobellieria bullata* and nowadays have been identified across arthropods [182]. Moreover, it was shown that they are conserved not only in amino acid sequence but also distribution in the CNS [182]. From different potential roles of SIFa in insects, of interest is that in *D. melanogaster* and *R. prolixus* these peptides were shown to regulate feeding [183]. This effect is similar to gonadotropin inhibitory hormone (GnIH) in vertebrates [184]. This is interesting, since the SIFaR was shown to be a homolog of the vertebrate GnIH receptor (GnIHR). GnIHR regulates food intake and reproductive processes and has been proved to stimulate feeding behavior and promote it over other kinds of behavior in mammals. However, it remains unclear whether the functions of the SIFamide- and GnRH signaling pathways are conserved across animal kingdom [184] but as a feeding related molecule might be interesting in studying fundamentals of obesity.

#### 2.12.4. Allatotropins

Allatotropins (ATs) are the multifunctional peptides, mainly participating in regulation of juvenile hormone (JH) biosynthesis, but also, their close relationship with feeding was confirmed. Recent research conducted on, for example, moth *M. sexta* or armyworm, *Mythimna separata* showed that deprivation of food enhanced expression of gene encodes AT [185,186]. Lee and Horodyski [185] also suggest that these changes are associated with reduction of energetically costly active ion transport across the larval midgut epithelium and reduction of energy consumption during starvation. In addition, the research performed by Konuma, Morooka, Nagasawa and Nagata [96] may indicate that ATs prolong feeding frequency. Moreover, it should be highlighted that AT can modulate contractile activity of insect gut, but the obtained results strongly depend on used insect model organisms. For example, Matsumoto, et al. [187] showed that administration of AT can decrease gut motility in *B. mori*. On the other hand, in *Helicoverpa armigera*, AT has myostimulatory properties [142,188,189,190,191,192,193,194,195,196,197,198,199,200,201].

#### 2.12.5. Leucokinins

Leucokinins (LKs) are identified till now in few invertebrate phyla, including insects [188]. It should be highlighted that, despite earlier suggestion, LKs and their receptor are not related to tachykinins and their receptors [188]. Current knowledge about physiological action of LKs in insects present that these neuropeptides modulate, for example, contraction of different insect muscles, including hindgut contractions, locomotion and fluid secretion by Malpighian tubules [188]. In addition, well-supported is the regulatory role of LKs on meal size, metabolism, and postprandial sleep [75,89].

Research conducted by Al-Anzi, et al. [189] showed that mutations in genes encode LKs precursor and receptor in *D. melanogaster* cause an increase in meal size. However, the meal frequency was reduced, for this reason the caloric intake was the same as that of wild-type flies. Further research conducted on this issue showed that LKs may regulate sucrose perception [190]. This is supported by the fact that activity of LK neurons is regulated by feeding, enhanced in response to feeding deprivation and reduced in response to glucose [190,191]. Despite participation in modulation of feeding behavior, LKs are also involved in regulation of digestion by inhibition of releasing of digestive enzymes from the insect midgut [192].

Like TRPs, LKs action on insect metabolism and food intake is linked with action of ILP signaling. The results obtained by Zandawala, et al. [193] showed that knockdown of genes encode LK precursor and receptor cause changes in the expression level of ILP-related genes.

#### 2.12.6. Limostatin

Limostatin (Lst) is a conserved 15-residue polypeptide hormone identified in *D. melanogaster* that suppresses ILPs secretion [194]. It is synthesized by APCs in *corpora cardiaca* mainly during starvation and regulated by carbohydrate restriction. When *Drosophila CG9918* gene, an orthologue of human neuromedin U receptors (NMUR), was knocked down in IPCs, the inhibitory effect of Lst on IPCs was abolished [194]. Moreover, when adult flies were *limostatin*-deficient, the hyperinsulinemic effect as well as increased content of triglycerides in fly body was noticed, and mutant flies were obese, and display phenotypes associated with insulin excess [194].

**Table 1:** Insect neuropeptides, its resemblance and potential usage as obesity model.

Insect Neuropeptide	Resemblance to Vertebrate Neuroendocrine System	Potential Usage as Obesity Model	References
Insulin-like peptides (ILPs)	Structural homology to insulin and insulin-growth factor and their receptors		[26,30,31,32,35,38,52,53,56,65]
	Insect ILPs are able to bind and activate human insulin receptors	Molecular basis of insulin signaling and sugar and lipid metabolism	
	Human insulin can activate insect ILP receptor	Participation of different components of insulin signaling in obesity development	
	The mode of action (including signaling pathways)	Interplays of different hormones in regulation of sugar and lipid metabolism	
	The physiological role		
Adipokinetic hormones (AKHs)	Physiological counterpart of mammalian glucagon	The role of glucagon signalization disturbance in development of obesity and metabolic disorders	[75,82,83,86,87,88,99,100,106,195]
	AKH receptors (AKHR) is a rhodopsin-like G protein-coupled receptor, which is related to the vertebrate gonadotropin-releasing hormone receptors	Hormonal regulation of lipid and sugar metabolism and interdependencies between hormones (glucagon, insulin, orexigenic factors)	
Corazonin (Crz)	Homologous of human gonadotropin-releasing hormone (GnRH)	The interactions between different neuropeptides and insulin signaling in development of obesity	[75,107,108,114,115,117]
		Influence of neuropeptides on food intake, starvation, and regulation of sugar level	
Hugin	Homolog of mammalian neuromedin U8	Participation of neuropeptides in taste recognition and feeding behavior in response to gustatory/nutrient signals	[118,120,121,123]
		Role of hormones in modulation of locomotion (including active food searching) and food intake	
Unpaired-1 (Upd1) and Unpaired-2 (Upd2)	Structural and functional analogues of leptin	Hormonal regulation of food intake and presence of satiety sign	[5,125,196,197,198,199,200]
	Resemblance of Upd1//Neuropeptide F dependencies to Leptin/Neuropeptide Y interplays	*Drosophila* with genetically induced obesity (e.g., upd1Δ or upd2Δ mutants) might be used as a good model for human obesity	
Allatostatin A and C (AST A and C)	Structural and functional homology to galanin (GAL) and its receptor	Participation of neuropeptides in regulation of feeding choices/decisions between different types of nutrients, which is crucial for balance of food intake and metabolic needs	[75,130,131,132,133,134,136,138,139,195]
	Structural and functional resemblance to somatostatin (SST)	Hormonal regulation of food intake and satiety	
Sulfakinins (SKs)	Structural and functional homology to cholecystokinin (CCK) and its receptor	Basis of signaling involved in modulation of sugar, protein, and lipid level	[52,140,142,143,144,145,148,149]
		Model for development of active substance based on SKs and CCK, which may lead to discovery of anti-obesity agent	
Short neuropeptides F (sNPF)	Structural and functional homology to prolactin releasing peptide receptor (PrPR)	Interplay of different neuropeptides with insulin signaling	[28,126,151,154,155]
		Due to orthology between PrPR signaling and sNPF, useful in research concern novel anti-obesity agents	
Neuropeptide F (NPF)	Structural resemblance to Neuropeptide Y (NPY)	Hormonal regulation of feeding choice	[125,126,157,158]
	Similar orexigenic action to NPY	NPF/Upd1 system a perfect target in studying obesity grounds, especially molecular basis of NPY and leptin signaling	
	NPF receptor could be activated by mammalian NPY		
Tachykinin-related peptides (TRPs)	Structural and functional similarity to vertebrate tachykinin (TKs)	Model organism in basis of participation TK signaling in development of the obesity	[11,89,99,159,161,201]
	Activation by Substance P (one of vertebrate TKs) insect TRP receptor	Molecular basis of lipogenesis	
		The interactions between different neuropeptides and insulin signaling	
CAPA-PVK neuropeptides	NMU homolog	Molecular basis of NMU-signaling in food intake and locomotor activity	[172,174,202,203]

## 3. Non-Peptide Hormones

Peptide hormones are the main axis of homology between insect and vertebrate neuroendocrine system. However, other insect hormones, like 20-hydroxyecdysone (20E) and JHs, also play an important role in the regulation of lipid metabolism and feeding behavior [204,205]. Ipso facto research concerning this issue may be helpful for understanding hormonal basis of lipid metabolism and obesity. Moreover, analogues of the mentioned hormones may be also use as potential anti-obese compounds [206]. The steroid hormone 20E play essential role in insect development, by modulation of molting [207]. However, current research also showed that this hormone participates in the regulation of wide spectrum of physiological events, including lipid metabolism. Research conducted, for example, by [208] showed that ecdysone signaling regulates lipid accumulation in *Drosophila* fat body, and knock-down of gene for ecdysone receptor cause increasing their storage. Moreover, research by Wang, et al. [209] and Tian, et al. [210] suggests that 20E may reduce food consumption which lead to lipolysis induced by the starvation and stimulate apoptosis in fat body of *B. mori*. It should be highlighted that action of 20E is closely associated with JH and ILPs. It is related to fact that synthesis and release of 20E and JH is regulated by the nutrients *via* ILP and target-of-rapamycin (TOR) signaling pathways [211]. Furthermore, [212] proved that 20E inhibits the expression of ilp2 and ilp6 genes, but upregulated ilp4 and ilp5 in mosquito *A. aegypti*. Interestingly, 20E may also elicit anabolic effect in vertebrate and may be a promise anti-obese factor. The results of recent studies showed that administration of 20E act preventively against hyperglycemia in insulin-resistant rats by decreasing hepatic glucose consumption [213]. In addition, as Foucault, et al. [214] showed, oral administration of 20E may reduce probability of obesity induced by high-fat-diet in mice. Moreover, 20E improve whole body insulin sensitivity in ovariectomized rats fed a high-fat-high-fructose diet (OHFFD) [206]. The second, very important non-peptide hormones participating in modulation of many aspects of insect life, starting from development and ending to vitellogenesis, are JHs [215]. Despite lack of vertebrate counterparts, the study concerning the role of JHs in regulation of lipid metabolism may be helpful for enrichment of our knowledge about hormonal basis of obesity. This is related to close relationships of JHs, ILPs, and 20E signaling [212]. Like 20E, this is related to the molecular pathway involved in the signal transduction and its modulation. In addition, in the case of JH, regulation of metabolism and some of the events associated with reproduction are mediated by the TOR pathway [216]. Research conducted by Maestro, et al. [217] and Perez-Hedo, et al. [218] showed that positive nutritional status or high sugar concentration may activate JH and vitellogenin production in insects, in which involved TOR kinase. As we mentioned previously, TOR pathway is also interconnected with the ILP signaling. Close relationships between JHs and ILPs are confirmed by [212] which showed that JH signaling regulate positively expression of *ilp2*, *ilp6*, and *ilp7* in *A. aegypti*. This process is mediated by Krüppel homolog 1 (Kr-h1) by direct interaction with promoters of these genes [212]. On the other hand, *ilp4* and *ilp5* genes are downregulated by Kr-h1. It should be highlighted that, Kr-h1 not only participate in the regulation of ILP signaling by JHs, but is also involved in the modulation of lipolysis by these hormones [219]. Interestingly this process in also mediated by, mentioned previously, FOXO transcription factor [219,220].

## 4. Other Factors

Another very important visceral organ which with proper functioning can be connected to obesity is the digestive system [221]. The specific cells of the gut of insects and mammals produce numerous peptide hormones, including the above-described neuropeptides F, CCH2, or TKs, thus regulating energy homeostasis [221]. Moreover, it was shown that gut epithelium produces a juvenile hormone-binding protein that promotes feeding [222]. This is why the insect gut, in connection to neuro-endocrine system, could be a gut source/model of potentially active molecules or a model to study basis of obesity [223]. The proper functioning of the intestine inter alia depends on the microbiota of the guts. The metabolites produced by bacteria such as short chain fatty acids (SCFAs) can regulate appetite, insulin signaling, and adipogenesis; therefore, the intestinal microbiota can direct its host to storage lipids in adipose tissue, leading to obesity [224]. It was shown several times in mice and was also proven in *D. melanogaster* [224]. For example, bacteria-free *D. melanogaster* were more likely develop hyperlipidemia, which is reversed after bacteria recolonization [224]. Recent studies also showed that exposure of *D. melanogaster* to sulfonamide antibiotics showed obesogenic effects correlated to lipid metabolism and changes is microbiota [225]. If the microbiome of the fly gut depends on the diet and contributes to the regulation of energy metabolism it can be a very usable model to study obesity promoting factors. Recently, not only *D. melanogaster* microbiota but also other models such as *A. mellifera* were proposed as a model to study host microbiota interactions [226].

## 5. Perspectives

In studies of the etiology, course, and treatment of human diseases for many years, vertebrates, especially mice, rats, and pigs were used [227]. Recent literature reports indicate that for this type of research, in terms of comparative and preclinical analyses, invertebrates, especially insects, can be successfully used [228,229,230]. Short life cycle, low breeding costs, and large number of offspring issued in short periods, as well the achievements of recent years, such as knowledge about genomes of several species of insects, and the development and refinement of genetic tools research methods are conducive to the use of these animals in a wide range of basic physiological, biochemical, genetic, and molecular researches [187,230]. Although there are fundamental differences between insects and mammals, including the morphology and anatomy of individual systems, the existing similarities in the structure, expression and regulation of gene activity, the course of signaling pathways, intercellular transport, as well as synaptogenesis or cells death induction mechanisms are rationale behind using insects as systems models in the study of human diseases [229,230] (NobelPrize.org). It is also important that the research conducted on insects is compliant with the so-called the 3R rule formulated by Russel and Burch [231] on the experimental methodology used, i.e., the reduction of the number used animals (reducing), use of alternative models (replacing), and improving methods research (refining).

The most-used model insect species in research on both the causes and the mechanism diabetes type 1 and 2, metabolic syndrome, and related abnormalities such as insulin resistance, and in testing of potential pharmacological agents’ effectiveness for therapy is *D. melanogaster* [230]. It is moreover justified as analysis of the genome has shown that 75% of all described human disease-related genes were found in *Drosophila* [232]. However, fruit fly is not the only species used so far in research of biomedical importance. Worth mentioning are *Tribolium castaneum* beetle, *Musca domestica*, *Calliphora vicina* flies, and locusts *L. migratoria* which are used in neurobiological experiments, and the *B. mori* silkworm for diabetes research [1,228,229,230]. In case of diabetes/obesity, it was found that in silkworm, *B. mori*, sugar level was higher after feeding a diet containing glucose [233]. It might lead to intensified sugar uptake and accumulation in the fat bodies, analogically as it is observed in mammalian liver and adipose tissue [233]. Injection of human insulin and AICAR (5-aminoimidazole-4-carboxamide-1-β-D-ribofuranoside), which act as an activator of AMP-activated protein kinase into hemolymph of hyperglycemic silkworm resulted, similarly as it is observed among mammals, in a decrease of sugar level in hemolymph [233]. In addition, Warbrick-Smith, et al. [234] postulates that consumption of energy-rich food might result in obesity occurrence. They showed that caterpillars of the diamondback moth *Plutella xylostella* which for multiple generations were reared on high-sugar-diet were able to eat excess carbohydrates without transforming it into fat which might be laid down [234]. *Libella pulchella* dragonflies are the next insect species which might be used in human obesity-correlated studies [235]. In those insects infected with Microsporidia and Apicomplexa, gregarine gut parasites, inability to lipid metabolism in muscles was observed, which was additionally shown as an increased level of carbohydrates in hemolymph [235]. The elevated concentration of glucose in individuals resulted in fat accumulation, which resembles mammalian obesity [235]. Moreover, researchers observed that short-term exposure to gregarine excretory products resulted in increased levels of blood carbohydrates and p38 mitogen-activated protein kinases (MAPK) activation in healthy individuals. Those observations in turn are characteristic for processes in mammals which are called metabolic syndrome [235].

Insects might be also used as a source of anti-diabetic substances like glycosaminoglycan isolated from dung beetle *Catharsius molossus* [236]. It was found that this compound reduced glucose, creatinine kinase, and alkaline phosphatase as well as cholesterol and TAG levels after application to obese, insulin-resistant mice, and mice with hyperinsulinemia [236]. Feeding high-fat diet rats with food with addition of glycosaminoglycan isolated from *Bombus ignites* caused changes in their hepatocytes [237]. Adding glycosaminoglycan induced inhibition of c-reactive protein activity and caused changes in and sero-biochemical parameters of phospholipids and free fatty acids, which indicated this compound to be potentially treatable for mammalian hyperlipidemia [237]. Peptides isolated from defatted mealworm *T. molitor* caused reduction of fat tissue weight, which is supported with results of gene expression pattern which showed that obesity-related membrane-associated protein levels which were in fatty acid transportation and utilization were up-regulated [238].

In addition, studies about usage of extracts from whole insects’ bodies were conducted [239]. Ethanol extracts of *T. molitor* larvae were tested on Mouse 3T3-L1 preadipocytes cell culture and resulted in reduction of lipid accumulation and triglyceride level in mature adipocytes [239]. In turn, adding of *T. molitor* larvae powder to high-fat diet mice in which obesity was pre-induced caused reduction of body weight and hepatic steatosis as well as aspartate and alanine transaminase enzyme levels [239]. All of these results indicated that insects-derived substances showed anti-obesity effects and were potential therapeutic agents for obesity treatment [237,239].

The presented results show the potential of usage of insect models in evaluating the therapeutic effects of anti-diabetic drugs, as well as knowledge of symptoms and ability to correlate them with similar symptoms in mammals, indicating that insects could be used as experimental models for studying biology and mechanisms underlying obesity in humans [233,235].
